# Service user involvement in mental health system strengthening in a rural African setting: qualitative study

**DOI:** 10.1186/s12888-017-1352-9

**Published:** 2017-05-18

**Authors:** Sisay Abayneh, Heidi Lempp, Atalay Alem, Daniel Alemayehu, Tigist Eshetu, Crick Lund, Maya Semrau, Graham Thornicroft, Charlotte Hanlon

**Affiliations:** 1 Department of Psychiatry, University College of Health Sciences, School of Medicine, Addis Ababa, Ethiopia; 2 King’s College London, Academic Rheumatology, Weston Education Centre, 10, Cutcombe Rd., London, SE5 9RJ UK; 3 Department of Psychiatry and Mental Health, Alan J Flisher Centre for Public Mental Health, University of Cape Town, Rondebosch, Cape Town, 7700 South Africa; 4 King’s College London, Institute of Psychiatry, Psychology and Neuroscience, Centre for Global Mental Health, London, UK; 5 Health Service and Population Research Department, King’s College London, Institute of Psychiatry, Psychology and Neuroscience, London, UK

**Keywords:** Qualitative study, Mental health, Ethiopia, Service user and carer involvement

## Abstract

**Background:**

It is essential to involve service users in efforts to expand access to mental health care in integrated primary care settings in low- and middle-income countries (LMICs). However, there is little evidence from LMICs to guide this process. The aim of this study was to explore barriers to, and facilitators of, service user/caregiver involvement in rural Ethiopia to inform the development of a scalable approach.

**Methods:**

Thirty nine semi-structured interviews were carried out with purposively selected mental health service users (*n* = 13), caregivers (*n* = 10), heads of primary care facilities (*n* = 8) and policy makers/planners/service developers (*n* = 8). The interviews were audio-recorded and transcribed in Amharic, and translated into English. Thematic analysis was applied.

**Results:**

All groups of participants supported service user and caregiver involvement in mental health system strengthening. Potential benefits were identified as (i) improved appropriateness and quality of services, and (ii) greater protection against mistreatment and promotion of respect for service users. However, hardly any respondents had prior experience of service user involvement. Stigma was considered to be a pervasive barrier, operating within the health system, the local community and individuals. Competing priorities of service users included the need to obtain adequate individual care and to work for survival. Low recognition of the potential contribution of service users seemed linked to limited empowerment and mobilization of service users. Potential health system facilitators included a culture of community oversight of primary care services. All groups of respondents identified a need for awareness-raising and training to equip service users, caregivers, service providers and local community for involvement. Empowerment at the level of individual service users (information about mental health conditions, care and rights) and the group level (for advocacy and representation) were considered essential, alongside improved, accessible mental health care and livelihood interventions.

**Conclusion:**

As Ethiopia increases access to mental health care, a fundamental barrier to service user involvement is beginning to be addressed. Our study identified further barriers that need to be tackled, including a supportive political climate, and receptiveness amongst stakeholders. The findings will inform the development of a model of service user involvement, which will be piloted and evaluated.

**Electronic supplementary material:**

The online version of this article (doi:10.1186/s12888-017-1352-9) contains supplementary material, which is available to authorized users.

## Background

Mental health service user and caregiver involvement is gaining ground as a core value in health policies of many countries [1, 2]. However, the extent of implementation varies substantially across countries, in part due to the complex, multi-dimensional and evolving nature of the concept of involvement [3, 4]. Involvement may occur at the ***micro-level*** (e.g. in individual care planning, assessment and care management), at the ***meso*** **-level** (e.g. in local service planning, monitoring and evaluation, advocacy, training and recruitment of staff, input into guidelines), at the ***macro-level*** (e.g. policy making, national level planning and advocacy) and in service-related research [1, 4]. In this paper, we focus on the active participation of local service users, caregivers, and its representatives in the mental health system components of policy making, service planning and deliver, advocacy, monitoring and evaluation, and research.

Service user and caregiver participation has the potential to contribute to mental health system strengthening through increased acceptability, relevance, appropriateness and efficiency of care, improved service quality and more positive attitudes of service providers [5–7]. In low- and -middle income countries (LMICs), service user and caregiver involvement has been proposed as an essential means of strengthening weak mental health care systems [8], to protect and promote service user rights and ensure successful scale up of quality mental health care [9–11]. In LMICs, service user and caregiver contributions to the mental health system have received minimal attention. Service users are often excluded from their rights to full citizenship and from meaningful participation in decisions that have a direct impact on their lives [12, 13].

In Ethiopia, most people with mental health problems do not have access to mental health care, with an estimated treatment gap (the number of people with mental illness who need treatment but do not receive it) of over 90% for severe mental disorders [14]. Lack of good quality care is associated with a high level of physical, emotional, economic and social suffering and disability [15, 16], excess mortality [17] and experience of physical restraint or other forms of deprivations of liberty, discrimination and abuse [18]. There is no mental health legislation to protect the rights of people with mental health problems [19] and there is limited representation for service users at the national level, with just one active advocacy group led by caregivers of people with mental health problems [20]. Nonetheless, at the national level there is commitment to improve access to mental health care through integration into primary care [21]. This provides an opportunity to increase engagement of service users and caregivers in service improvement as service development and expansion proceeds. However, there is a lack of evidence on the best models for successful involvement of service users and caregivers in LMIC settings [12].

This study was conducted as part of the ‘Emerging mental health systems in low- and middle-income countries’ (Emerald) project, which investigates the health system requirements for successful scale-up of integrated mental health care in six LMICs (Ethiopia, India, Nepal, Nigeria, South Africa and Uganda) [22, 23]. The aim of this study was twofold: to explore the experiences, perceived barriers and facilitators to service user and caregiver involvement in mental health system strengthening; and to inform development of a scalable model of involvement for Ethiopia.

## Methods

The authors approached the study from a phenomenological stand-point to explore service user and caregiver involvement from the perspectives of the participants. The study design was a qualitative study using in-depth interviews with key stakeholders.

## Study setting and context

The health care delivery system in Ethiopia is structured into three levels of care: primary (primary hospital, health centres and health posts), secondary health care (general hospitals) and tertiary (specialist services) [20]. A primary hospital provides services to about 100,000 people. A rural health centre with five satellite health posts serves approximately 25,000 people. The community is linked to each health facility and participates actively in the health system through the innovative, community-based Health Extension Program and Health Development Army [20].

This study was conducted at both the national level and in districts around Butajira town in the Gurage Zone, Southern Nations, Nationalities and Peoples Region of Ethiopia. Butajira has been a community-based mental health research site for over 20 years, including a large population-based study of people with severe mental disorders [14]. Linked to mental health studies, a psychiatric nurse-led out-patient mental health service was established in 1997, located in Butajira Hospital [18]. In the neighbouring district of Sodo, a district level plan for mental health care integration into primary care is being implemented and evaluated as part of the PRogramme for Improving Mental health carE (PRIME) [24, 25]. PRIME had not started to provide mental health care at the time of this study. More than 85% of people in the Gurage Zone reside in rural areas and are reliant on subsistence farming. Small-scale trading is common in the urban settings and cash crops (e.g. chilli peppers, khat and papaya) are sources of cash for the rural people in the area.

## Participants

Thirty–nine key stakeholders were selected purposively to participate in the study. At the national level, three planners/policy makers (PP) (two from the Federal Ministry of Health, one from the World Health Organisation) and four psychiatrists involved in policy, planning and/or service development were approached and interviewed by co-authors CH and AA. The national level participants were included because of their experience of working in mental health policy making and planning and/or intimate knowledge of the mental health system. At the district level, one district health administrator (DHA), eight primary care facility heads (HCH), service users (SU) with clinician-confirmed diagnoses of severe mental disorders (schizophrenia, schizoaffective disorder, bipolar disorder or major depressive disorder with psychotic features) (*n* = 13) and their caregivers (CG) (*n* = 10) participated. The district level professionals were included because of their familiarity with the health system at the local level and because of their involvement in managing primary care service delivery. Service user and their caregivers were included based on their gender, religion and duration of experience receiving biomedical mental health care. The service user and caregiver participants were approached initially by the district health service providers and asked if they would be willing to speak to project data collectors about possible participation in the study. In all cases, the service users were in remission or stable and able to give informed consent.

## Data collection

Data gathering was through a face-to-face in-depth interview with each participant. A topic guide was developed by last author (CH) for the Emerald cross-country study and adapted for the Ethiopian context on the basis of experiential knowledge of co-authors (CH and AA). The interview guide explored service user involvement in relation to aspects of the mental health system (policy making, mental health planning and service development, mental health research and evaluation of mental health services) in terms of: (i) experience of service user/caregiver involvement, (ii) how service users/service user organizations might contribute, (iii) barriers to involvement and (iv) suggestions about interventions needed to facilitate service user and caregiver involvement. During data collection, probing and clarifications were used. The topic guide was developed iteratively as data collection proceeded, for example, expanding to ask respondents about service user involvement more generally in the health system as so few respondents had experience with respect to mental health care. The interviews with national informants were conducted in English by two co-authors (CH and AA). All of the district level interviews were conducted in Amharic, the official language of Ethiopia, by co-authors TE and DA. The interviews with service users and caregivers were carried out at Butajira mental health research office. The interviews with heads of health centres were conducted in a private facility. The interviews lasted an average of fifty five minutes. District level participants were remunerated for their time and transportation costs. The interviews were audio-recorded with prior written consent from all participants.

## Data analysis

The interviews were transcribed verbatim in Amharic by experienced transcribers, and TE and DA translated into English, with the first author (SA) cross- checking selected audio files and transcripts for accuracy before coding. Data analysis was done using a thematic analysis approach [26]. Open Code 4.02 [27], a qualitative software computer programme, was employed for the textual data analysis [28]. SA familiarized himself with the data by repeatedly listening to the audiofiles and reading through the transcripts. Initially SA and CH coded four transcripts independently and compared the coding schemes and developed a draft coding framework. A further two transcripts were coded by SA and CH independently and consensus was reached. SA coded the remaining transcripts using the existing codes and adding further codes where relevant, with close supervision by CH. Sub-themes and themes were derived from the primary codes following further cross-checking by SA and CH and further comments of other co-authors (HL, CL). The final themes were developed deductively, based on the basic topic guide questions, and inductively by adding themes that emerged from the data (see Additional file 1). A comparative analysis was made between the categories of respondents. Illustrative accounts were identified (see Additional file 2).

### Validity checks

Single counting of the number of participants endorsing particular perspectives was used as a means to increase validity [29]. Other validity checks included cross-checking emerging themes against the data and efforts to seek out deviant cases [30].

### Reflexivity

The interviewers of the policy makers and planner stakeholders were psychiatrists with PhDs in mental health epidemiology. The interviewers for the district level participants were Masters level research assistants from Addis Ababa University; one male and one female. When interviewing non-literate participants from the rural areas, some interviewees appeared to defer to the views of ‘experts’, as has been observed previously with a similar population [31]. Efforts were made to communicate to participants that their views and perspectives were equally valid, that any information they disclosed would be confidential and that it would not have any bearing on their health care. Although care was taken to only interview people with severe mental disorders (SMD) who were not acutely unwell, some respondents were not able to tolerate lengthy interviews. The involvement of researchers with diverse educational backgrounds (psychiatry, psychology, sociology, demography and epidemiology) broadened the interpretation of the data.

## Results

### Socio-demographic characteristics

The national level respondents, district level health administrator and health centers heads were all male. The district level respondents were either health officers with degree level training, or nurses at degree or diploma level. The socio-demographic characteristics of the service user and caregiver participants are presented in Table 1.

**Table 1:** Socio-demographic characteristics of service user and caregiver participants

Characteristics	Service users	Caregivers
Number of participants	13	10
Gender		
Male	8	4
Female	5	6
Age (years)		
≤ 25	0	2
25–34	2	3
35–44	6	0
45–59	4	3
60+	1	2
Level of Education		
Unable to read or write	5	2
Informal education	3	1
Primary education	4	5
Secondary education	1	0
Certificate and above	0	2
Religion		
Muslim	5	2
Orthodox Christian	8	3
Protestant Christian	0	5

The analytical framework included four key themes related to involvement: (i) experience of involvement, (ii) barriers to involvement, (iii) potential benefits, and (iv) capacity building needs for greater involvement of service users and caregivers in the local mental health system.

### Experience of involvement

All groups of participants indicated that there was almost no involvement of service users and caregivers in mental health system domains. The national level participants noted that service user and caregiver involvement in policy making and planning was extremely limited. As one respondent commented:

*PP: There is only nominal participation, the ‘user ‘association is at best promotional and no meaningful attempt is being taken by the Ministry of Health to engage them.*

*Policy-maker/planner ID3*

At the primary health care level, people with mental health problems and their caregivers were not represented and their direct involvement in health service and system activities was non-existent.

*HCH. We invite for participate the “One to Five” community organization networks to report general health problems; we don't specifically enquire for mental health issues....*

*Health Centre Head ID1*

The service user and caregiver participants also reported that they had no experience of involvement in mental health system domains. Some service user and caregiver participants reported experience of being the subjects of research. Most considered being approached and their involvement as a research respondent to be valuable for themselves as well as for the improvement of the mental health service.

*I: But do you think it is important in any way to involve people with mental health problems in research?*

*SU: I do think so. I am pleased you are here to listen to what I have to say because most of the time most people are not willing to listen to what we have to say because they believe we are mentally ill. … But it blesses me to have people who listen to my ideas around.*

*Service user ID*

Some service user and caregivers had concerns about the relevance of research to recipients of care. The current research approach appeared to be top-down with limited knowledge of how the findings would benefit them.

*CG: …Yes, many students from universities made researches but nothing is obtained out of it.*

*Caregiver ID5*

*They [researchers] came from Amanuel hospital [the only psychiatric hospital in Ethiopia]. Just like you [the interviewer]. Maybe, you came from the branches… Things move from the stem to the branches. Not from the branches to the stem. People from there will call me here or come to my house for the study. They [the researchers] will discuss many things, though it is not implemented.*

*Service user ID5*

### Barriers to involvement

**Fig. 1:**
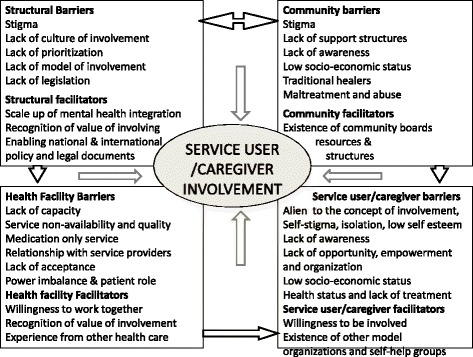
Conceptual model of barriers and facilitators to service user involvement in mental health system strengthening

All the participants (39/39) in this study stated that there were many barriers to service user and caregiver involvement in the mental health system. The barriers to involvement are summarized in a multilevel conceptual framework encompassing the structural/system, community, health facility and individual service user/caregiver levels, along with potential facilitators to involvement (see Fig. 1).

#### Involvement as an alien concept

Most of the service users (11/13) and caregivers (6/10) were new to the concept of involvement, and repeatedly asked “what do you mean”, “what is that?” in response to questions exploring their experience of involvement in policy-making and planning, service development and quality monitoring. Commonly, they struggled to give examples of how they might contribute to mental health system strengthening, and many (10/23) instead focused on their role as a patient. Some service users (3/13) considered involvement as an assigned role, which they would be willing to embrace as a sense of duty, rather than as a right or benefit. They did not see involvement as the role of a service user and considered health system issues to be the responsibility of government workers.

*Service User (SU): Quality assurance kind of thing is done by higher bodies or by people assigned by the government for this purpose. …*

*Interviewer (I): So, don’t you think that your participation improves the service delivery?*

*SU: You [service user] will add nothing, since this [worker] is appointed by the government.*

*Service user ID5*

Some of policy-making/planning level participants reported that there was no culture of involvement of service users and caregivers at the mental health system level; as a consequence there was no structure or model for how involvement might work in practice. When asked about service user involvement, one planner replied:

*Policy/planner (PP): I think that's an excellent initiative. I see that it is very difficult to implement that in Ethiopia. I think there's not that type of culture in Ethiopia so I don't know how workable it is. Policy-maker/planner ID6.*

#### Stigma and mental health status

Participants (18/39) spoke in many different ways about how stigma and discrimination serve as barriers to service user and caregiver involvement. Stigma and exclusion were noted to operate within the health system, in the community and even to affect the self-identity of the person with mental illness and their family. Half of policy/planning level participants (4/8) perceived that service providers, policy makers and health system managers at all levels had negative attitudes towards mental health and people with mental health problems. Examples presented by some interviewees (14/39) to support this perspective included (i) the lack of prioritization of mental health in the policy agenda compared to other public health concerns, and (ii) the widespread assumption that service users would be unable to contribute anything of value to the mental health system because of the effects of mental illness. Some policy/planning level participants (3/8) articulated that system-level stigma would be an insurmountable barrier to service user involvement.

*PP: Yes, absolutely stigma is contributing.*

*I: Within policy-making and planning?*

*PP: I mean, for me, it is hard to separate anything you know. You are dealing with human beings who are doing the planning; you don’t just put a new hat on when they come here. It is part and parcel of the whole value system. Mental health, mental illness, has been neglected, stigmatized type of diseases and of course, it is the same person who is stigmatizing mental illness who is working in planning…*

*Policy-maker/planner ID1*

Some policy/planning level participants (2/8) doubted the possibility of service user and caregiver involvement at a higher strategic level, but recommended empowerment of service users and caregivers at the community level.

The low status of people with mental illness in society was considered to be an important barrier to involvement. The service user and caregiver participants (10/23) described repeated experiences of unsupportive, discriminatory behavior from the local community and a lack of acceptance of their right to take on social roles. As a consequence some interviewees (6/23) spoke of experiencing diminished opportunities for productive lives and exclusion from their civic rights (employment, participation in meetings, and voicing their say). Exclusion resulting from stigma was also reported to affect their access to treatment and thereby to impede recovery and limit their capacity to be involved in system strengthening. Two participants spoke passionately about this issue:

*Currently, there is no a good thing towards mental health patients in the society, once a person gets sick mentally, the society discriminates and takes that person as useless; they don't think mental health problem can be treated and the patient can be better and live a normal life again.*

*Health Centre Head ID8*

*Are you a fool? Only our families know what our problem is, but the others don’t care. ... only you may want to help us or understand what our problem is; otherwise they wish us to disappear …uhh…nobody wants us.*

*Service user ID10*

Many of the service users and caregivers (11/23) also had low expectations of their own capacity to contribute; some service users (3/13) preferred instead that their caregivers speak on their behalf. Other participants (6/39) also expressed the view that caregivers would be better placed to be involved in system strengthening due to their freedom from mental ill-health.

*To negotiate and to participate in planning, he [service user] should be healthy. How can a mentally ill person participate in management activities? Disabled people can do this since they are physically disabled. But mentally ill person faces difficulty on the main part of thinking.*

*Service user ID5*

### Lack of resources and empowerment

Lack of resources and empowerment of service users/caregivers, and other stakeholders in the mental health system were also underlined (17/39) as barriers to involvement. A number of participants (14/39) spoke about the need to provide support (financial, space, organization, and training) and facilitate empowerment of service users and caregivers at the grassroots level. Some of the service users and caregivers (6/23) indicated that their low educational level, low socio-economic status and livelihood problems were major factors in their lack of involvement in mental health systems.

*It is because of our status…are educated and uneducated people equal?*

*Service user ID12*

During the interview process, both service users and caregivers (15/23) were noted to be reluctant to express their views, particularly in relation to their potential contribution to system strengthening for example, saying *“we agree with what you told us”*, *“everything you said is important”*, and that they were *“not the expert”*. Furthermore, participants (12/39) expressed the view that service users and caregivers are not mobilized for involvement, empowered, organized into groups or represented in strategic decision making. As a consequence, they lack power and support, and are reluctant to ask for their rights.

*We never speak for our rights, we have fears...Our fear is…it is difficult to speak about something which the society doesn’t understand and nobody gave us strength to go forward other than giving a small amount of money and other things.*

*Service user ID9*

#### Poor access to mental health care

Poor access to adequate mental health care was considered to be a major barrier to involvement of service users. The service user and caregiver participants (8/23) reported that they had to walk long distances, or incur high transport costs, and spend a lot of time seeking mental health care from the centralized, specialist service. Some caregivers (4/10) also noted the logistical challenge of conveying a person with mental illness (for example taking person to the health facility, medication use, follow-up). As a consequence not all people could access treatment.

Service users and caregiver participants (9/23) noted problems in service delivery, including waiting time, medication provision, and service providers’ lack of professional behavior and competence.

*They [service providers] don't treat mental health patients properly, the ill-treatment and abuse must be corrected, and the people at the health centre must be disciplined in case of mental health patients. They should be caring, should consider the mental health patients just like their children, and loving attitude.*

*Caregiver ID9*

Some service users and caregivers (5/23) spoke of feeling uncomfortable to express any form of criticism of the people providing them with mental health care, due to fears that this could jeopardize their access to a scarce and valued resource.

*SU. We have a fear.*

*I. What type of fear?*

*SU. They (health service officials) use to call us anything and they are trying to help us at least so it is difficult to talk about their deficiencies.*

*Service user ID10*

### Potential benefits of involvement

A number of potential benefits of involving service users and caregivers were reported, grouped into two sub-themes: (i) advocacy, fighting exclusion and improving service quality, and (ii) awareness raising and service promotion.

#### Advocacy, fighting exclusion and improving service quality

Most of the participants (21/39) across all stakeholders talked about various contributions that service users and caregivers could make, including (i) advocacy for better physical health services, (ii) suggestions of integration of non-medicinal interventions, (iii) help in reducing waiting times, (iv) assistance in service standards improvement by providing information about their experiences of the extent and adequacy of the service provided. Many participants across the participants groups (14/39) also considered that service users and caregivers could strongly demand improved accessibility of mental health services, service expansion, budget allocation, service inclusiveness, efficient utilization of resources, and to bring their unique lived experiences to focus attention to mental health care during health system planning.

*We can oversee/push how the allocated budget is spent, whether they [health centre heads] are spending it properly for the intended purpose or not, because we can't be sure unless we participate there.*

*Caregiver ID6*

*It [involvement] has a significant impact on the improvement of the service quality and inclusivity, as you know in our country many strategic plans come from above … if you base your planning… the low level, or the users, first of all there will not be any wastage and outflow of resources… if we [Health Centre Heads] involve the patients and their caregivers, in the planning, and even in the future research, then our plans would be very effective and problem solving.*

*Health Centre Head ID5*

Some participants from all groups (7/39) considered that improved participation would contribute to better understanding and support for service users. One service user (1/13) remarked *“the one who knows how comfortable the bed is, is not the person who made the bed but the person who slept on it” *(*Service user ID10*) to illustrate the importance of sharing their experiential knowledge with service providers. Many of the service user and caregiver participants (10/23) also suggested that service users and caregiver involvement can help to protect patients from maltreatment, voice the rights of service users and improve service providers’ behaviors.

*Well, I think it [involvement] will be important because it will help people with mental health problems to have control about the quality of service they receive and manoeuvre the way their problem is addressed. It [involvement] can also help protect people with mental health problem from any abuse and maltreatment. Their [service users and caregivers] participation could also mean that the professional can get needed information from them about their need and situation.*

*Service user ID3*

#### Awareness raising and service promotion

Study participants (11/39) underlined the important role that service users and caregivers could play in raising awareness and mental health service promotion through experience sharing, including providing testimonials about mental health care and how they are living with mental health problems productively. About half of the service users and caregivers reported (12/23) their willingness to share their own experience of mental illness and living with the illness, about the medicine and treatment, the improvement in their health following treatment, and to create awareness about mental health services in the local community.

*Our[caregiver]contribution will be...we will tell other patients and caregivers to go to hospital...I use to tell everyone to go there and get treatment...there are many people with this problem on the streets and who are walking around...*

*Caregiver ID8*

Some participants (8/39) spoke of how service users and caregivers can communicate first hand with the wider local community to raise awareness more effectively than health workers, who tend to talk about mental health in the abstract. For example three health centre heads (3/8) expressed:

*… if that patient is treated well he will be witness and will publicize positively and propagate the good result of the program to the community, the treated-patients would spread out where they took the medicine and what type of program helped them restore their health. In addition the participation of the patients or their caregivers will be a good source of constructive comments, on the both strength and weakness of the program.*

*Health Centre Head ID8*

### Need for capacity building

Participants (23/39) highlighted the following as essential capacity building strategies to support greater service user involvement: (i) enabling community structures and past experience, (ii) mental health advocacy and (iii) the need for service use/caregiver mobilization and empowerment.

### Enabling community structures and past experience

Many participants (20/39) emphasized the importance of giving recognition for developing the mental health system, particularly planning, at the lower district level. This was informed by the needs of service users to improve service quality, to make the service inclusive, to plan health service resources more efficiently and to receive feedback and correct mistakes at a higher system level.

*I: But if that [involving service users and caregivers] were possible, do you think that could be constructive?*

*PP: Not could be, it should be. Unless you involve the users, unless you involve the beneficiaries, how do you know? For me, it is very, very critical. And some day it is going to come, but it requires awareness, organization and stuff like that. I think it is very important.*

*Policy-maker/planner ID1*

The service users and caregiver participants (5/23) identified existing structures which could be leveraged to promote involvement, including availability of health workers at the grassroots level, opportunities from frequent social gatherings, and structures for awareness creation and mechanisms for selection of a representative who could voice their interest in involvement. An opportunity for learning from experiences in other aspects of health care (e.g. reproductive health) and HIV was noted.

*…About HIV, they are doing many things in different organizations, in health facilities and also on media; so, they need to do better than HIV, you know mental illness can be treated like HIV; therefore, it needs everyone's participation.*

*Service user ID11*

#### Mental health advocacy

Across the different groups of participants (16/39) the need for mental health advocacy to overcome stigma and discrimination was emphasized.

The participants noted the lack of knowledge about mental health and mental health services and linked this to abusive practices. One caregiver (1/10) commented the following:

*We need to educate the families, the caregivers and the community altogether, not to physically and psychologically attack mental health patients. People used to insult, condemn, despise mental health patients, they may be even severely attacked to death, if they leave their village, they cannot participate in a normal social relations, this will aggravate their anger and touches their emotion…*

*Caregiver ID10*

Some service users (3/13) underlined the need for empowerment in their livelihoods and support from non-governmental organizations, pointing at the success of this strategy in the field of HIV/AIDS. The different groups of participants (17/39) expressed a need to be equipped with training in different areas including: how to work with others, communication skills, social aspects of mental health, and how to care for patients. The health centre head participants (6/8) emphasized the need for training and one of the health centre heads (1/8) recommended the following areas:

*To work with them [service users and caregivers] you have to have better knowledge. This is because they may raise their real experience since they are living with the problem. They may ask you actual issues which they face when they took the medicines. If you can clarify them [what they need] they will build confidence to work with you. You need to know more. The kind of training that helps you [healthcare providers] to work with people and help you to convince others are the important ones.*

*Health Centre Head ID8*

Some service user and caregiver participants (5/23) also indicated the importance of training for service providers in the areas of care-giving and treatment for patients.

### Service user and caregiver mobilization and empowerment

Interviewees across all groups (12/39) expressed that service users’ mobilization and empowerment are the appropriate area for greater involvement in the mental health system and other domains of life, such as social roles. The participants outlined various benefits of having a representative organization, but also raised practical concerns about the need for space for meeting and representation in the formal structure of the health system for involvement, a relevant strategy and guidance to support different stakeholders to work together:

*To bring change, we [service users] should get together and discuss about solutions and things which are helpful for us…We should be together, we need to stay together as we couldn’t stand problems related to our sickness, so, we need to organize or we should establish our own unity; we want our health…, they [health service managers] should tell us the rules and regulation from the government and also things we should do not only to oppose their work.*

*Service user ID10*

*…In that case my first recommendation even before giving them [service users] financial and any material support is [to] establish a kind of club, where they can discuss together ... then education can be conducted on different subject matters … and in this way we[caregivers] can provoke them[service users]to stand for their right and the rights of patients. In this way we can also reduce the problems…Yes! We need training. We need to know what we have to do, at all level, so that we will have acceptance by the people [community] whom we are going to work together. ... We must know the extent and the limits of our rights and what duties we have to carry out.*

*Caregiver ID10*

A need for support with transport and financing of a representative organization was also raised by some service users (5/13).

## Discussion

In this qualitative study from Ethiopia, we examined systematically the perspectives of a range of stakeholders about the possibilities for service user and caregiver involvement in policy making, planning, service delivery, mental health research, monitoring, and scale-up of mental health care. Only a few previous studies from LMICs have focused on service user involvement in policy development, and most focused on involvement in self-help groups, individual care plans or as ‘subjects’ of research rather than system level involvement [12, 13, 32].

Although starting from a low baseline, most stakeholders in this study considered mental health service user involvement to be an important and achievable goal of the health system. Participants identified a range of potential benefits from service user involvement. Service user and caregiver respondents also anticipated individual benefits of being more closely involved in system issues, including access to basic information about mental health conditions and treatments, advice on the best way for them to provide care and a feeling of recognition. These potential benefits of involvement have also been identified within the limited existing publications from LMICs [13, 33].

Despite the recognition of benefits, few respondents had personal experience of service user involvement in any aspects of mental health system strengthening. Low levels of involvement have been reported even from better-resourced LMICs with more empowered and mobilized service user groups [12]. In our study, a number of barriers to achieving service user involvement were identified, as well as potential facilitators (see Fig. 1).

The barriers to service user/caregiver involvement will now be discussed in relation to potential strategies to promote service user involvement: (i) creating an enabling environment, (ii) multi-system approach to mental health advocacy and fighting stigma, (iii) comprehensive mental health service in primary health care, (iv) ensuring human rights for greater involvement, and (v) service users/caregiver mobilization: organization and empowerment.

### Creating an enabling environment

The study participants noted that there was no specific strategy or model to guide how best to involve service users and caregivers, and a lack of clarity about the roles and responsibilities of the different parties. There are similar results from LMICs as well as high income countries [2, 12]. In a systematic review of studies from LMICs [12], there was almost no evidence and no conceptual framework to inform effective involvement of service users and caregivers. Similarly, in a recent narrative review from high income country studies (1969–2016), most attempts at involvement were criticized for exclusivity and for being tokenistic, with little evidence on how to support involvement of a diversity of service users and members of the public, rather than a few selected individuals [2].

Many high income countries where service user involvement is embedded have clear policy provisions and legislative requirements about service user/caregiver and public involvement [2, 6, 34]. The Ethiopian national constitution [35] clearly guarantees the rights of people with disabilities; and the country has ratified the United Nations Convention on the Rights of Persons with Disabilities [36]. However, the Health Policy [37], National Mental Health Strategy [21] and the Health Sector Transformation Plan [20] do not include explicit provisions articulating how service users and caregivers should be involved at the level of the mental health system. There is no separate national human rights review body with authority to oversee mental health facilities and to ensure service user rights; there is also no legislation to protect persons with mental health problems against discrimination [19]. These enabling frameworks need to be put in place in the future revisions of the Health Policy, National Mental Health Strategy and Health Sector Transformation Plan to institutionalize and guide service user involvement.

### Multi-system approach to mental health advocacy and fighting stigma

In our study, negative attitudes towards people with mental health problems were reported to be pervasive and a significant barrier to service user/caregiver involvement. Within the mental health system, despite high level political commitment, a lack of (i) prioritization of mental health, (ii) access to mental health services, and (iii) representation and support for the empowerment of service users, was identified. At the health facility level, service users and caregivers are poorly informed about their rights, the nature of their illness, available treatments and services, and may experience negative attitudes and abusive behaviour. At the societal level, people with mental health problems often experience discrimination and maltreatment; individually mental health problems may affect their sense of identity and self-worth, exacerbating disempowerment and impeding realization of their rights.

These findings are similar to reports from studies conducted in LMICs and globally. In a recent narrative review, Semrau et al. [38], concluded that “*Stigma and discrimination have been identified as major negative forces against full citizenship and social participation everywhere that they have been assessed*”. Similarly, in a qualitative study from South Africa [13], stigmatization and low prioritization of mental health, poverty,and incomplete recovery and community support were identified as major barriers to involvement. A study from India [39] also showed various impacts of stigma, including social exclusion, restricted opportunities in civic rights, impaired quality of life and avoidance of mental health services due to fear of labelling. Such structural stigma undermines access to mental health care, civic rights such as education and employment, and affects all aspects of daily living, contributing to disempowerment, and feelings of hopelessness, helplessness and guilt about being a burden to others [40, 41].

International narrative and conceptual reviews have identified a lack of evidence on anti-stigma interventions to support service user involvement, particularly in LMICs [38, 42, 43]. Nonetheless, service user and caregiver involvement in anti-stigma interventions is considered to be an important principle for mental health system development [42]. Studies also recommended lessons about the important roles of service users and caregivers in (i) advocacy to mental health and services, (ii) informing policies and research, (iii) giving testimony about their mental problems and services with a lasting effect on reducing stigma [44, 45]. Despite this strong evidence, there seems to be little commitment to promoting and supporting service user and caregiver involvement, particularly in LMICs [38, 42, 45].

### Comprehensive mental health services in primary health care

The service user/caregiver participants in this study spoke of accessible and adequate mental healthcare as being a priority for them, and recommended expansion of care to include psychosocial support and rehabilitation services. All three groups who participated in the study articulated the need for capacity building, promotion of awareness-raising and facilitation of structures for service user and caregiver involvement. A situational analysis study on the challenges and opportunities for integrating mental health conducted in Sodo district [46] identified various difficulties, including financial constraints, high level of poverty, low literacy, social deprivation, limited level of community awareness, high level of stigma and abuse, absence of health system structures and support systems for mental health care, and a lack of reliable supplies of medications among others. However, concerted efforts are now made to implement multi-faceted mental health care plans in Ethiopia, which in turn provides an opportunity to integrate service user involvement [47].

The Ethiopian National Mental Health Strategy states that the single most important factor to improve the situation of people with mental health illness and caregivers who are experiencing stigma, discrimination and human rights abuses is to increase the availability of mental health services [21]. The integration of mental health services into primary health care needs to consider the social, economic and educational status of service users, as well as the stigmatizing and discriminatory practices that tend to disempower and marginalize service user and caregiver involvement [48, 49]. There is emerging evidence that interventions to improve access to mental health care combined with promoting livelihoods and peer support (e.g. the BasicNeeds model of mental health and development) can lead to empowered service users and caregivers who are able to take on active roles within society, and regain social capital and influence [48, 49]. For example, Raja et al. [48] in their case study of the BasicNeeds model implemented in Nepal found considerable evidence of service user and caregiver involvement in income generation activities and productive work. In rural Kenya, Lund et al. [49] evaluated the effects of participating in the BasicNeeds program on the outcomes of mental health including social support, and poverty alleviation of a cohort of people living with severe mental disorders. Their results showed substantial and statistically significant improvements in mental health, quality of life, social functioning and economic activity after two years.

### Ensuring human rights for greater involvement

There is a need to address the stigmatizing and discriminatory practices that hinder the involvement of service users and caregivers in the mental health system, as well as in their full participation in social life and realization of their civic rights [50, 51]. This approach is focused on redressing the unfair distribution of power and discriminatory practices, with an emphasis on empowering service users and caregivers to know and claim their rights, and building the capacity and accountability of individuals, organizations and professionals to promote respect, protection and fulfillment of responsibilities [33, 50, 51]. The human right-based approach is guided by core values and principles of participation, accountability, non-discrimination, empowerment and legality and requires health facilities, goods and services to be available, accessible and acceptable services that help service users and caregivers to exercise their rights to health [50, 51].

### Service users/caregiver mobilization: Organization and empowerment

Participants of this study recommended organization and empowerment of service users and caregivers, particularly at the grassroots level, referring to what has been achieved by the government and non-governmental organizations to empower and support similarly stigmatized service users with other long-term health conditions (e.g. HIV/AIDS associations). Other studies also found that mental health service users are invisible, poor, voiceless and comprise a fragmented movement that may result in weak mental health advocacy, particularly in LMICs [8, 51, 52].

Addressing the multi-level barriers to greater involvement of service users and caregivers in the mental health system and other domains of life requires inputs of various stakeholders (national to local level service providers in all government structures, development agencies, non-governmental organizations and self-help organizations). These stakeholders can create opportunities to access resources, training and skill development, health and psychosocial support and provide space and structures (strategies, rules, legislations) [45, 52, 53]. In addition, ensuring national and international human rights instruments for the protection of the rights of service users; enacting comprehensive anti-discrimination legislation with robust enforcement mechanisms are also key areas that need attention [52, 54]. Empowering service users to self-organize and advocate for their interests and needs promotes their recognition and develops their confidence, strengths, resources and skills [52, 53]. Empowerment of service users also ensures a collective voice to influence and lobby for policy and legislative reforms [51]. In a study of seven African countries, networks of service users were found to play a range of important and influential roles, including serving as alternatives to traditional mental health services that deliver only medical treatment, development of income generation opportunities and building service users’ work skills, provide psychosocial support, active participation in advocacy, lobby for improved government services and support to build self-esteem in resource poor settings [53].

### Strengths and limitations

The strength of the current study lies in its use of qualitative research methods with a wide range of participants who brought broad perspectives on service user and caregiver involvement across the mental health system. Our findings should be considered within the limitations of the study. As a qualitative study, the findings may not be generalizable to broad populations of service users and caregivers because our study focused on the views of people with severe mental disorders. The professionals who facilitated service users and caregiver recruitment may have been selective in recruiting participants and participants who were not contacted may have different perspectives.

## Conclusions and research implications

Service user and caregiver involvement is almost non-existent in the Ethiopian mental health system. Multilevel stigmatizing attitudes, discriminatory practices and lack of capacity impede service user and caregiver involvement in mental health system strengthening, civic rights and social roles. The planned mental health care scale-up through integration into primary health care will address one of the fundamental barriers to service user involvement and provides a critical opportunity to institutionalize involvement. The findings of this study will inform the participatory development and piloting of a model of service user and caregiver involvement in the new integrated primary mental health services in Ethiopia.

## Additional files

Additional file 1: Table S1. Themes, Sub-Themes and Codes. (DOCX 17 kb)

Additional file 2: Table S2. Code queries. (DOCX 73 kb)

## Abbreviations

AIDS: Acquired immunodeficiency syndrome
CG: Caregiver
DHA: District health Administrator
Emerald: Emerging mental health systems in low- and middle-income countries
HCH: Health Center Head
HIV: Human immunodeficiency virus
LMICs: Low- and middle-income countries
PP: Policy maker/Planner
PRIME: PRogramme for Improving Mental health carE
SMD: Severe mental disorders
SU: Service user
