# Supplementary material for: Service user involvement in mental health system strengthening in a rural African setting: qualitative study
Source: BMC Psychiatry. 2017 May 18;17:187. doi: 10.1186/s12888-017-1352-9 (PMC5437561; doi:10.1186/s12888-017-1352-9)
Supplement: Supplementary file 1 — Themes, Sub-Themes and Codes. (DOCX 17 kb) [file 12888_2017_1352_MOESM1_ESM.docx]

Table S1: Themes, Sub-Themes and Codes

| Themes | Sub-themes | Codes |
| --- | --- | --- |
| Experience of involvement in mental health System | No involvement in mental health system | No experience of involvement  Understood at individual level |
|  | Limited involvement in mental health system | Limited involvement  Concern about value of research  Concern about research procedure |
| Barriers to involvement in mental health system | Alien to the involvement concept | Difficulty conceptualizing concepts of involvement  New about involvement  Lack of /no expectation of involvement  Involvement as assigned role  Lack of model /structure of involvement  Lack of participatory approach |
|  | Stigma and mental health status | Ascribed patient role  Health status  Doubt about service user involvement  Lack of prioritization  Lack of acceptance  Lack of community support  Lack of opportunity  Paternalistic caregivers  Perceived lack of capacity  Perceived negative effect  Stigma(attitude)  Stigma(discrimination)  Stigma(knowledge problem)  Stigma(self)  Stigma(structural) |
|  | Lack of resource and empowerment | Lack of ability to articulate  Lack of association/organization  Lack of SU/CG representation/say  Lack of empowerment  Lack of resource  Lack of opportunity  Low education/knowledge/awareness  Low self-esteem/capacity |
|  | Poor access to mental health care | Lack /Unavailability of accessible service  Power imbalance  Reluctant to criticize  Issues about medicine  Issues to be improved in service  Concern about competence /behavior of service providers  Medicine only services and concern about asking more  Time off work |
| Potential benefits of involvement | Advocacy , fighting exclusion and improving service quality | Advocacy role  Budget allocation and control  Demand creation  Demand for right to be involved  Make service accessible /service expansion  Demand for services more than medicine  Experiential support  Unique lived experience  Fighting stigma  Patient protection  Improve service  Knowledge/information source  Improve providers behavior |
|  | Awareness raising and service promotion | Awareness creation  Case finding  Mental health promotion |
| Need for capacity building | Mental health advocacy and stakeholder empowerment | Advocacy to overcome mental health stigma  Empowering stakeholders to involve  Need to equip with training |
|  | Need for SU/CG mobilization and empowerment | Need for SU/CG organization and representations  Space and structure for involvement  Treatment and more services in addition to medicine |
|  | Enabling community structures and past experience | Existing social structure for awareness raising  Experience from other health care user  Experience from other healthcare user associations  Experience of public involvement in health system  Right to be involved  Value  SU/CG  lived /experiential knowledge  Valuing SU/CG involvement |
|  | Mental health advocacy | Advocacy to overcome mental health stigma  Empowering stakeholder to involve  Need to equip with training |
|  | Service user and caregiver mobilization and empowerment | Challenges to organize and select representative  Criteria to select representative  Space and  structure for involvement  SU/CG Organization and representation  Treatment & more service in addition to medicine |
