# Supplementary material for: Service user involvement in mental health system strengthening in a rural African setting: qualitative study
Source: BMC Psychiatry. 2017 May 18;17:187. doi: 10.1186/s12888-017-1352-9 (PMC5437561; doi:10.1186/s12888-017-1352-9)
Supplement: Supplementary file 2 — Code queries. (DOCX 73 kb) [file 12888_2017_1352_MOESM2_ESM.docx]

*Table S2: Themes, sub-themes, codes and illustrative quotes*

| ***Themes*** | ***Sub-themes and corresponding codes*** | *Illustrative quotes* |
| --- | --- | --- |
| ***Experience of involvement in mental health system*** | ***No involvement in mental health system***   - *No experience of involvement* - *Understood at individual level* | *I: What is the extent of patient caregiver participation in mental health planning?*  *…*  *R: Till now they didn’t participate in planning.*  ***Health Center Head ID2***  *I: Have you ever been participated in mental health service planning before?*  *P: No, I didn’t.*  *I: Have you ever been participated in mental health service quality control?*  *P: No, I didn’t.*  *I: Do you think participating patients in mental health quality assurance is important?*  *P: Yes.*  *I: What will they contribute?*  *P: They will control quality, follow our wellbeing, help people who have mental retardation and they will give us medicines on time…..*  *I: Have you ever been participated in this kind of study?*  *P: No.*  *I: What about in the hospital?*  *P: No, I didn’t. They asked us whether we feel better now or not.*  *I: You are getting services which were previously planned. What do you think if you participate in the planning process?*  *P: We do not have any problem if we have meetings. We can* ***enjoy ourselves and refresh our mind****. When we discuss about good things,* ***we will renew our selves****.*  ***Service user ID6***  *I: Have you ever been participated in mental health planning or other studies?*  *P: I didn’t participate in any study, I only get treatment.*  *I: Have you ever been asked to participate in studies may be from the ministry or the university?*  *P: Nobody asked me to participate.*  *I: Do you think that patient and caregiver participation is important in planning or researches?*  *P: Yes, I think this is good. I want to be cured. I want to participate. I want to see changes in my health.*  ***Service user ID4*** |
|  | ***Limited involvement in mental health system***   - *Limited involvement* - *Concern about value of research* - *Concern about procedure of research* | *I: Ok, emm, just keeping on the idea of talking about planning what about service user involvement in planning?*  *R: It is very limited, extremely.*  *I: for mental health*  *R: Yes*  *I: What about for other disorder, health conditions?*  *R: Very limited*  ***Policy-maker/planner ID1***  *I: What is the extent of service user participation in the planning and delivery of mental health policies and plans at a national level?*  *R: There is only a nominal participation, the ‘user ‘association is at best promotional and no meaningful attempt is being taken by the ministry of health to engage them.*  ***Policy-maker/planner ID3***  *I: Have you ever been participated in planning before?*  *CG: Yes, we started treatments in this hospital around 1987E.C. before 19 years. Since then many people from Addis Ababa University came to make researches and we were participated in many of the interviews. When I or my father took my mother to the hospital they often ask us questions. This is one form of participation since the information we gave may used as an input for their study…..In my opinion, I can say that we have been participated in researches for a long time.*  *I: As you told me before you participated in many studies, do you remember which kind of studies they were?*  *CG: Yes, we participated in many studies. For example, in one study researchers call us and collect samples of hair, blood and the like of mental health patient. The other study which I remember is that they call caregivers and make some interview. They asked us about the patients’ condition whether there is mental health problem in the family or not.*  *I: When you participate in studies how much is your level of involvement? I mean, which way did you participate, by oral interview, by filling questionnaire or what?*  *CG: There were times which we made written interview. They give us questionnaire to be filled by us and these were about our living condition, about the patient and about so many things. I was asked to fill questionnaire two times.*  ***Caregiver ID3***  *I: Do you think that these kinds of studies do not have relevance?*  *CG: Yes, many students from universities made researches but nothing is obtained out of it.*  ***Caregiver ID5***  *I: Where did they come from?*  *P: They came from Ammanuel hospital. Just like you. May be, you came from the branches. I know you are not from here. Things move from the stem to the branches. Not from the branches to the stem. People from there will call me here or came to my house for the study. They will discuss many things though it is not implemented. For example last time we discussed and some people request a plot of land to build a house. They also added that if they get their own house they will be fine.*  ***Service user ID5*** |
| ***Barriers to involvement in mental health system*** | ***Involvement as an alien concept***   - *Difficult to conceptualize* - *New about involvement* - *Lack/no expectation of involvement* - *Involvement as assigned role* - *Lack of model/structure of involvement* - *Lack of participatory approach* | *I. Your contribution...what could be your specific contribution if you can participate there?*  *CG. What do you mean?*  *I. How can you help?*  *CG. Whatever they ask*  *I. For example?*  *CG. I use to think, whether it is financially or in other aspects.*  ***Caregiver ID8***  *I: Have you ever been participated in the hospital’s plan preparation for mental health patients?*  *CG: No, they didn’t invite us to participate.*  *I: You told me that you didn’t participate in mental health policy preparation but if you would participated what kind of contribution can you make?*  *CG: I don’t have anything to say. What do you mean by contribution? Is that about health or?*  *I: About mental health?*  *CG: About mental health?*  *I: I mean the hospital planes, if you get a chance to participate, what kind of contribution can you made?*  *CG: I don’t know. I am illiterate. Actually, I can give a response for what I am asked, not more than that.*  ***Caregiver ID5***  I*. You have been with this problem for 12 years and you knew the difficulties you faced, so, if they invite you to participate in the planning process, how can you contribute as a person who suffers a lot?*  *P. Is it financially....we are going to pay by selling our maize or wheat/ whatever we have.*  *I. It may be based on what you are able to, starting with sharing of your opinion?*  *P. I know only taking my medication per month; I came here today because of your invitation.*  *I. So, what should be done?*  *P. You are the one who knows what things should be done.*  *I. There are things I may know, but what we need is to know what your opinions, needs and desires. There information are very helpful for the betterment of the service such as the medication you are receiving now. Because you know many things, you know how much you suffered, you know what type of care and support you need too, just like other types of patients, on top of that you can also tell us what you feel about the reactions of other people towards you?*  *P. I don’t know anything*  *I. So, how would you feel about the idea of participating in programs regarding mental health service improvement?*  *P. I don’t know anything other than taking medication.*  *I. But you should know some things?*  *P. I know nothing*  ***Service user ID8***  *I: For example in supplying medicines, or in any other service which you want to be included. Till now somebody was planning for you, what if patients and care givers participate in this area?*  *CG: They will give medicine when I need and injections when I need. I don’t know any other thing.*  *I: What do you thing in the future to be improved?*  *CG: I couldn’t say add this or avoid this. I don’t know more than this.*  *I: In your opinion, what kind of support is necessary to improve the services that you want to get?*  *CG: I don’t know. We are old, we don’t know.*  ***Caregiver ID1***  *I. Do you think that you are able to evaluate the performance of health facilities regarding provision of medication and their service delivery in general?*  *P. I don’t think so.*  *I. Why?*  *P. You know what;* ***I don’t think it is our responsibility****.*  ***Service user ID9***  *I: Do you think participating patients in mental health quality assurance important?*  *P: Quality assurance kind of thing is done by higher bodies or by people assigned by the government for this purpose. If you went to other health center, they will not show you good face. But here they will care about you.*  *I: So, don’t you think that your participation improves the service delivery?*  *P: You will add nothing, since he is appointed by the government.*  ***Service user ID5***  *I: If there are no models for mental health care, what about for other health conditions?*  *R: I think when it comes to service user participation, the other areas are also in the same boat with mental health, and this is an indication of the overall lack of* ***participatory approach in Policy development****, this true for all public policy!*  ***Policy-maker/planner ID3***  *I: Okay. Another component of planning, which is done in some countries, is to involve patients or caregivers or their representative bodies. How do you see that happening in Ethiopia if at all? And what's your view on that?*  *R. I think that's an excellent initiative. I see that that is very difficult to implement that in Ethiopia. I think there's not that type of culture in Ethiopia so I don't know how workable it is.*  ***Policy-maker/planner ID6*** |
|  | ***Stigma and Mental health status***   - *Ascribed patient role* - *Health status* - *Doubt about service user involvement* - *Lack of prioritization* - *Lack of acceptance* - *Lack of community support* - *Stigma(attitude)* - *Stigma(discrimination)* - *Stigma( knowledge)* - *Stigma(self)* - *Stigma(structural)* | *I: What is the extent of patient caregiver participation in mental health planning?*  *R: Here there are some gaps between patients and physicians and also between caregivers and physicians.*  *I: What is that gap?*  *R: Physicians thought that if a person is mentally ill he couldn’t be cured and back to his normal condition. But as we said before if the service is delivered at the primary level the awareness of the people around the service will be improved….If I became aware of these things, I will teach other under my level.*  ***Health Center Head ID2***   1. *Have you ever involve in policy making [mental health care]?*   *P. After I have been sick I never been involved whether in policy making or in a meeting.*  *I. Really?*  *P. No, because* ***I am sick, nobody is accepting*** *what I am speaking, people use to say I have no proper expression.*  *I. Why?*  *P. If you are a sick person…uhh…I had been unemployed for a long time even after I got better.*  *I. why?*  *P. It was hard for me to find a job because most people* ***think of me as a sick person****, but I prayed so hard and God helped me to get a job after a long time.*  *I. Are they (the people) think of you?*  *P. Yes, they do.*  ***Service user ID9***  *I: I guess if you go back fifteen years, HIV would have very much been like that. Quite stigmatized and marginalized. So how do you think they got from where they were then to where they are now? Is there something that could be applied to mental health or do you see mental health as different in Ethiopia?*  *R: This is a difficult question, I don't know whether the paradigm related to HIV scared policy makers and I don't know. But of course it's much more prominent; it's a much more prominent issue than mental health. In the public agenda I mean.*  *I: So you're implying more of the barrier might be from sort of the policy side rather than from the patient/caregivers side? Is that?*  *R: Yeah.*  *I: Okay. Do you think it's something worth pursuing or testing out or piloting in the Ethiopian context?*  *R: In principle yes.* ***I don't know if it is viable****. When sometimes I discuss this with some colleagues who are sometimes very experienced psychiatrists, I see that there's a lot of, I have the feeling that they're very skeptical about this and that the overall mentalities is that what people with mental disorders think doesn't really matter to what we do. So that is not really the best approach to promote such initiatives. When the mental health legislation was being reviewed there were no people who represented persons suffering from mental disorders, there were family members, etcetera, etcetera. And when I raised the issue I was asked what is the added value of the people who have mental disorders? So, that was the position of the Ministry of Health. So this doesn't give us a very rosy scenario.*  *I: So that would be high level kind of planning. But then what about sort of maybe going down lower and still at the systems level but maybe sort of thinking more about quality improvement? Do you think it could work maybe at the lower level where it wouldn't work at the high level?*  *R: It might but I don't have a strong opinion on this and I'm afraid also that lay persons share the same mentality as policy makers.*  *I: Okay. So it's sort of stigmatizing attitudes is that a good way to put it?*  *R: Yeah, I'd say so.*  *I: Do you think the stigma is part of the reason mental health is not given its due priority part within policy?*  *R: No it is not a stigma.*  *I: where would stigma fit? Is it contributing at all? Do you think?*  *R: Yes, absolutely stigma is contributing.*  *I: Within policy-making and planning?*  *R: I mean, for me, it is hard to separate anything you know. You are dealing with human beings who are doing the planning; you don’t just put a new hat on when they come here. It is part and parcel of the whole value system. Mental health, mental illness, has been neglected, stigmatized type of diseases and of course , it is the same person who is stigmatizing mental illness who is working in planning, so…,*  ***Policy-maker/planner ID6***  *I: But what about would you see a role of family members or patients at sort of a more high level, like planning, being involved in decisions about how we develop the services, how we monitor them at a kind of higher level. Would you –*  *R: Well, higher level may be too much, but if we kind of bring that kind of service at the lower level, that base of the society. If you look at the pyramids, that are the base, right? [reference to WHO pyramid] So if we empower the base, then for a high-level policymakers life becomes much easier because in the end of the day, it's the base, the community that does much of the job*  ***Policy-maker/planner ID*5**  *I. Can the society accept you?*  *P. The others might not accept even though I want*  *I. Why is that?*  *P. only the health professionals (psychiatric), how can the others accept us?*  *I. Why?*  *P. How can they accept?*  *I. Why not?*  *P. Are you a fool? Only our families know what our problem is, but the others don’t have care. People in our community are not like people in the other part of the country, they wish patients to disappear but on the contrary people in other places try to help patients as much us they can, it is true.*  *I. Why?*  *P. They use to think us, as unable to speak /think, don’t have stands…uhh…no one is accepting /trusting us because we are patients; we are helping ourselves by taking our medication properly, forget the others.*  *I. So, what are the barriers….*  *P. Others are not interested; only you may want to help us or understand what our problem is otherwise they wish us to disappear …uhh…nobody want us.*  ***Service user ID10***  *I. What is the importance of your participation?*  *P. Currently, there is no a good thing towards mental health patients in the society, once a person gets sick mentally, the society use to discriminate and take that person as useless; they don't think mental health problem can be treated and the patient can be better and live a normal life again. If a person get sick mentally, they use to take him/her to traditional hillers (religious), and then if that person couldn't be better, they use to assume he/she is useless. So, it will be useful if we can participate in such programs and create awareness in the society about mental health and its treatability like other diseases.*  ***Health Center Head ID8***  *I. What is the attitude of the society toward mental patients?*  *P. Do you mean the other people?*  *I. Yes! I mean the other people?*  *P. Don’t ask me about the other people, what can I tell you?*  *I. What is it?*  *P. It is very hostile and very bad. It is shameful when we saw them sick, they will be disregarded, everyone dishonors them, and people did not consider that they can be sick sometime tomorrow. They aggravate the sickness they don’t care. They shout calling by the name then they exaggerate everything by their bad words, but other people looked with enmity and unfriendliness.*  *I. Do you think there will be recognition and acceptance if the families of mental health patients and the patients themselves speak on behalf of mental health patients?*  *P. How could they find recognition and acceptance? No, it will not be.*  *I. For example! Do you think people will respond positively if a mental health patient speaks what type of treatment and need they deserve from their society?*  *P. You mean if they speak?*  *I. Yes!*  *P. No! They will not accept.*  *I. Why?*  *P. Because they don’t believe the mental health patient can act properly, even if they improve or recover they don’t believe the mental health condition can be treated.*  ***Caregiver ID8***  *I: For example if you organize yourself, it would help you to get better service or drugs. Forming an association helps you to have a representative and increase your negotiation power with the government during planning and implementation.*  *P: To negotiate and to participate in planning he should be healthy.* ***How can a mentally ill person*** *participate in management activities? Disable people can do this since they are physically disabled. But mentally ill person faces difficulty on the main part of thinking*  ***Service user ID5***  *I: where would stigma fit? Is it contributing at all? Do you think?*  *R: Yes, absolutely stigma is contributing.*  *I: Within policy-making and planning?*  *R: I mean, for me, it is hard to separate anything you know. You are dealing with human beings who are doing the planning; you don’t just put a new hat on when they come here. It is part and parcel of the whole value system. Mental health, mental illness, has been neglected, stigmatized type of diseases and of course , it is the same person who is stigmatizing mental illness who is working in planning, so…,*  ***Policy-maker/planner ID1*** |
|  | ***Lack of resource and empowerment***   - *Lack of ability to articulate* - *Lack of association /organization* - *Lack of SU/CG representation/say* - *Lack of empowerment* - *Lack of resource* - *Lack of opportunity* - *Low education/knowledge/awareness* - *Low self-esteem/capacity* | *I. What do you think the reason would be?*  *P. It is because I was a sick person…uhh… I used to speak trivially and people who saw me at that time still assume I am the same person.*  ***Service user ID9***  *I: Do you have mental health patients association?*  *P: We haven’t.*  *I: Don’t you have an association.*  *P: We didn’t yet establish an association.*  *I: What about the national mental health association?*  *P: We didn’t meet and discuss about this issue. Do you mean like disable people’s association?*  *I: What is that?*  *P: Disable people are organized themselves and the government helps them to support their living. Actually here there is a big difference between disable people and mentally ill people. A disable person may have a problem on some part of his body but a mentally ill person has a problem on his brain, the main part of his thinking. So it is difficult for us to form an association.*  ***Service user ID5***  *I: You are many in number here. Do you have an association?*  *P: We don’t.*  *I: Do you gather and discuss on issues when you meet?*  *P: No, we don’t.*  *I: Really?*  *P: Yes.*  *I: You only go to facilities, get medication and back to home.*  *P: Yes, we only went for medication and we do not make any meeting.*  *I: Don’t you have an association or any kind of organization?*  *P: We don’t have any kind of organization.*  *I: As I told you before mental health planning are made in the ministry of health. Do you think participating patients in planning is important?*  *P: In my opinion, it is important. Organizing ourselves and having discussions will give us moral and refresh our mind.*  ***Service user ID6***  *I: What is your view on that in relation to expanding mental health care?*  *R: I think is wonderful like in the …., I had worked in the director of several programs and it was mandatory to work with the user group, the advocacy group. But it takes a lot of sophistication to do that especially… what we have is the national Ethiopian mental health society. Very weak. The families. But, you know I do believe, definitely, it is also our responsibility to help them organize but we are not doing a good job. We are not doing anything actually….*  *I: So what do you think might be holding it back, perhaps awareness might be holding it back?*  *R: Awareness, lack of sophistication, I mean.*  *I: What do you mean by that?*  *R: Well, in other words, the families and the user groups are not sophisticated and it is not encouraged. It requires resources. But you know in the ….what they did were they provided these types of entities with financial resources to organize. It takes some resources to organize for this group. You know, they need to meet, they need paper, and they need a little bit of an office… it is just not easy… and also the stigma. The stigma really doesn’t allow you to say ‘I am mentally ill, let’s get together and advocate. You are mentally ill too’…..*  ***Policy-maker/planner ID1***  *I. What can hold you to participate in the evaluation process or what type of problems can you face?*  *P. We might not have the information about when and where the evaluation is going on….there will not be other problems.*  ***Service user ID12***  *I: Have you ever been participated in the hospital’s plan preparation for mental health patients?*  *CG: No, they didn’t invite us to participate.*  *I: You told me that you didn’t participate in mental health policy preparation but if you would participated what kind of contribution can you make?*  *CG: I don’t have anything to say. What do you mean by contribution? Is that about health or?*  *I: About mental health?*  *CG: About mental health?*  *I: I mean the hospital plans, if you get a chance to participate, what kind of contribution can you made?*  *CG: I don’t know. I am illiterate. Actually, I can give a response for what I am asked, not more than that.*  ***Caregiver ID5***  *I. You are the service users, but how can you evaluate the service you are receiving?*  *P. We are not controlling what are they are doing or what they are giving us.*  *I. My question is what if you can control?*  *P. We couldn’t till now, the government is just helping us, we are illiterate people we don’t certainly know.*  ***Service user ID10***  *I. What make you to develop this type of doubts?*  *P. It is because of our status…are educated and uneducated people equal?*  *I. Is it because you are illiterate?*  *P. I think it is like that.*  ***Service user ID12***  *I. What do you think will be the main obstacle for you or your participation not to contribute whatever you say to me until now and other things you may provide for the effective implementation service?*  *P. For my participation?*  *I. Yes!*  *P. May be my age, may be my educational background, and mental status or conditions and the like and his capacity to speak*  ***Caregiver ID10***  *I: In your opinion what kind of support do you think is important? You have told me that you believe the participation of people with mental health problem is important. It could be both from the policy analysts and the patient’s part; do you think there are barriers for people with mental health problem and their caregivers to actively participate in planning and policy formulation activities?*  *CG: Well, most of the time people with mental health problem are shy and fearful. They have low self-esteem so they don’t approach the professionals to express their ideas and opinions.*  ***Caregiver ID4*** |
|  | **Poor access to mental health care**   - *Lack/unavailability of accessible service* - *Power imbalance* - *Reluctant to criticize* - *Issues about medicine* - *Issues to be Improved in the service* - *Concern about competence/ behavior of providers* - *Medicine only service and concern about asking more* | *I. What is the extent of patients and caregivers participation in the planning of mental health service?*  *P. There is no service to do that.*  ***Health Center Head ID6***  *I: Is there anything that should be improved, especially when you came to the hospital to get mental health service?*  *CG: Yes, there is a problem. We live far away from this hospital, so we are expected to travel longer distance to get the service. We get the medicine every two or one month. I bring my daughter may be once or twice a year for checkup.*  ***Caregiver ID1***  *I: From which village did you come?*  *P: From Sedabira.*  *I: How far is Sedabira from here?*  *P: It is a five hour walk from here.*  *I: Five hours for each way? Five hours to come and five hours to return?*  *P: Yes.*  *I: Isn’t there any health center around where you live?*  *P: There is one but mental health care service isn’t part of the service they give.*  *I: They don’t provide health care service?*  *P: No, they don’t.*  *I: What compromises a quality health care service for you? For example what if the service is made available at the health care center in your village? Which one do you think is convenient for you? Here or there?*  *P: it would have been convenient if I could get the service there because it is closer to where I live. It would have saved me the transportation cost. Because it is far from where I live, I take the medicines for three to four months at once. Once I have taken three hundred and this other time four hundred pills at once because it is far from where I live. If it was closer to where I live I could have taken my drugs every month or so and do follow my checkups.*  ***Service user ID3***  *I. Therefore, the Ministry of Health have decided to integrate mental health service in to primary health care centers just like many other health services, what do you think if you are allowed to involve officially in the, planning, evaluation and implementation of this program? And what do you contribute?*  *P. This is very excellent idea. It is very difficult to understand the suffering we have lived, it is different for those who have not even experienced, to wait for our turn, to take them to the hospital and many other things but if it close, we can take them, they be given some pain killer, they can follow up their medical from nearby,*  ***Caregiver ID10***  *I: I have finished my questions; do you have anything to add?*  *CG: It is better to give the service in the nearby health center since some places are very far from Butajira hospital. The physicians give a fixed number of medicines which is enough for one month. If there is a waste you do not have anything to replace. You will wait until that month is completed without medicine. This will create inconsistency in taking the medicines.*  ***Caregiver ID3***  *I. What do you need to raise your voice and ask, even though the society thinks like this we are patients like other patients and also our sickness can be treated, so, the government should see us equally with other patients and deliver care for us like it does for others?*  *P. We never speak for our rights, we have fears.*  *I. What type of fear?*  *P. Our fear is….it is difficult to peak about something which the society doesn’t understand and nobody gave us strength to go forward other than giving a small amount of money and other things.*  ***Service user ID10***  *I. I think they are in shortage of medication...do you think that you can prevent such type of problems by telling them what is happening in the health facilities?*  *P. Yes, if we can identify what the problems are, they will be careful due to the evaluation [and its consequence] but everybody is afraid [nobody is courageous to do that].*  *I. Why?*  *P. They might not treat us properly.*  *I. Do you think they will forbid you?*  *P. Yes, they might; there are many problems here.*  *I. So, do you think that exposing the problems during the evaluation can bring change, if you are not afraid of the backfire?*  *P. Yes!*  *I. How?*  *P. As I told you before, it might be difficult to get the service if we expose them; so, it is always holding us back and also the nature of the sickness[mental illness] doesn’t allow to do that, let alone this we have even sacrificed a lot but we never succeed. ….*  *I. Do you mean the patients?*  *P. There are many problems regarding the service delivery even though we can’t speak about it, one day I brought him here and I asked the person [health professional] who was in the office to give us medication but he walked out without saying a word, even though I begged him by telling how I suffer to brought him here*  ***Caregiver ID7***  *I: Do you think that giving trainings to improve mental health service for health service providers is important?*  *CG: Yes, it is very important.*  *I: In which case do you think it is important?*  *CG: They should be trained how to care patients; how to treat patients; how to avoid things that aggravate patient’s mental health problem.*  *I: Yes, it is correct. What about on the quality assurance part?*  *CG: Making the physician to arrive on time and to be available in working time. The patients should also come on time. For example if the patient arrive after working time and claim for the service, this doesn’t work. So the patient and care givers should aware of these things ahead of time.*  ***Caregiver ID2***  *I. You have told me that previously your comments on the mistreatment of maternal health care was corrected, and in the same way what can you say about improvement of the mental health service delivery?*  *P. They don't treat mental health patients properly, the ill-treatment and abuse must be corrected, and the people at the center must be disciplined in case of mental health patients. They should be caring, should consider the mental health patients just like their children, and loving attitude,*  *I. Do you mean during the diagnosis?*  *P. Yes! They should handle the patients slowly and carefully, so that the mental health as well as mentally retarded patient will feel friendly*  *I. What are the major problems around the health care professionals, Hospitals and other health workers?*  *P. In fact, treating and diagnosing mental health patients need to be tolerant and companionate, the physicians will suffer a lot, because they don't understand fully and I don't know what they have to do but I think they should be careful and considerate.*  ***Caregiver ID9***  *I: What things should be added to make the service better off?*  *CG: We are at the lower class; we came here; they gave us the medicine and we don’t have any talk with service providers. We didn’t lose anything.*  ***Caregiver ID1*** |
| **Potential benefits of service users and caregivers involvement in mental health system** | **Advocacy, fighting exclusion and improving service quality**   - *Advocacy role* - *Budget allocation and control* - *Demand creation* - *Demand for right to be involved* - *Make service accessible/service expansion* - *Demand for services more than medicine* - *Experiential support* - *Unique lived experience* - *Fighting stigma* - *Patient protection* - *Improve service* - *Knowledge/information source* | *I. …Yes. So, what contribution you would have if you participate with the government officials (they can be Kebele or Woreda officials)?*  *P. They will let us to participate if they need anything from us.*  *I. In which cases can they need you? Or what can you contribute?*  *P. We can oversee/push how the allocated budget (by the government or by NGOs) is spending whether they are spending it properly for the intended purpose or not, because we can't be sure unless we participate there.*  *I. Okay, you can participate on budget part, how about on the service delivery?*  *P. About the service delivery, we can put pressure to deliver a better service everywhere.*  ***Caregiver ID6***  *I. What do you think about patient or the caregiver can contribute to the effectively of the program?*  *P. I have already told you,…., if that patient is treated well he will be witness and will publicize positively and propagate the good result of the program to the community, the treated-patients would spread out where they took the medicine and what type of program helped them restore their health and in addition the participation of the patients or their caregivers will be a good source of constructive comments, on the both strength and weakness of the program.*  ***Health Center Head ID7***  *I. The one who faced the problem is the one who know the difficulty, so, you knew the problem very well and the difficulties you face, then what are you going to advice the planners or what contribution could you have?*  *P. Our contribution will be...we will tell other patients and caregivers to go to hospital...uhh...I use to tell everyone to go there and get treatment...there are many people with this problem on the streets and who are walking around...uhh...people who can be better if they have caregiver...I use to think like this about them...but I am not letting my son to go to street until the day I die, I am going to let him get treatment and I have a hope that he will be fine.*  ***Caregiver ID8***  *I. So, just like that if you participate in health care planning with health care planners, do you think your participation would bring any change?*  *P. Yes, it may not be particularly to my own health, but to my husband and other patients and caregivers,* ***I can speak on their behalf, I can participate for the wellbeing of the patients and their families.***  *I. What can you tell for the planners, what can you contribute about the service delivery?*  *P. I can tell the day of treatment for people who are in need, I can promote the service.*  ***Caregiver ID9***  *I. So what will be the particular benefit of your participation to the policy making?*  *P. to indicate what has to adjusted, to show the concern for example whenever there is a need to increase the drugs we increase, what about to decrease, if there are medication without drugs, to open a space where they can enjoy, to create a place where they can learn or normalize their life, because that can also facilitate some rest for us [caregivers], because we don't let them to partake in our regular social activities, we don't let them to be in the shop, or other places we use to tie them and keep them separate.*  *I. Are you recommending a rehabilitation centre for them?*  *P. Yes! Something like recreational, play centre, I can recommend something like that will develop their mind thinking.*  ***Caregiver ID10***  *I. How it is important?*  *P. Its importance is, for example you are not going to feel it if you don’t know what the problem like and I believe people on the street with this problem can be fine if they are able to get treatment, I never insult or attack them back even though they usually do such things to me because I was there and I know what it’s like. I can take them to the health facility by giving them cigarette, tea and bread for them I know mental health problem make adults like a child; and I can explain to the health professionals there what that persons problem and how much treatment they need, and also I can show them myself as an example how much the treatment helped me; there are many people who didn’t get that chance and live on the street*  ***Service user ID9***  *I. Do you think that your participation or asking them to change medications and telling them what type of service should be delivered contribute for the improvement of the service delivery?*  *P. Yes, they are not saying no if we go and discuss with them and we have obligation to go and talk to them because we are the victim. For example,* ***the one who knew how much comfortable the bed is not the person who made the bed but the person who slept on it,*** *so, as long as we are the victims and the people suffering from the problems we should go and tell the professional how we are suffering from the medications, that is our obligation….uhh…*  ***Service user ID10***  *I. How can you contribute in a different way than others?*  *P. Other people and we (patients) are not the same, for example, if I see a patients on my way I will search where that person lives and who are his/her families and then help to find health care service.*  *I. Why are you different?*  *P. As I told you, I have been there, for example, if other three people and me see a person with this problem, the other three may not understand that person’s problem like I do. I will talk to him/her why is he/she get angry and acting like this, and then I will find that persons family and tell them to let her/him get treatment.*  ***Service user ID11***  *I: How could patient or caregiver participation improve how mental health care is implemented in this zone / region?*  *R: They could participate in awareness creation so that patients in the community seek help and utilize services. They could also help with fighting stigma and discrimination. They should take responsibility in monitoring quality of services by means of offering feedback of the services they received and availability of medications, etc. I feel very comfortable to work with them in this way. Perhaps, I might need training on how to organize family groups /user groups*  ***Policy-maker/planner ID2***  *I: Mental health patients themselves …it could also be you….do you think it is important if you could take part in, for example, service quality assurance and controlling committees or other task groups? Do you think it will be helpful to improve the standard of quality of the service you are receiving if you could work together with the professionals?*  *P: Well, I think it will be important because it will help people with mental health problem control the quality of service they receive and maneuver the way their problem is addressed. It can also help protect people with mental health problem from any abuse and maltreatment. Their participation could also mean that the professional can get needed information from them about their need and situation.*  ***Service user ID3***  *I. Do you think patients or caregivers participation can improve the service delivery ...?*  *P. Yes, it will increase the quality too.*  *I. Why?*  *P. Because, there might not be a condition for them to ignore when they see people with this problem, and also they might indicate for those people where they can get the service, so, I believe their participation can bring a better thing.*  *I. Do you think that patients’ participation in the discussions like what type of service should be delivered can improve the service delivery?*  *P. Yes, I do. Firstly to let them participate in the discussions will help to develop their awareness better, second on the basis of the information collect from them, we can understand what things has been carried out and what things are jumped or ignored, as well as what major problems they faced from the community.*  *I. Do you think they can comment to improve the service delivery?*  *P. Yes, I think they know better than anyone; although they can’t be better than the professionals; they might work hard by taking it with responsibility because they suffered a lot from it.*  ***Policy-maker/planner ID8***  *I. So, how could their participation contribute for the improvement of service quality?*  *P. It has a wider contribution. Because the first and foremost they are the starting and the ending points,….uhhh…. so if the community is aware of the cause, the effect, the prevention and the precaution of the illness then they can control it.*  ***Health Center Head ID6***  *I. As I told from the beginning, PRIME works on the service delivery whereas EMERALD is engaged in building the system and support the structure, so it is good to know which components are useful, who are participated, therefore, from your own experience and perspective, how important is the participation of the patients and their caregivers in the planning, policy making and quality improvement of the service?*  *P. It has significant impact on the improvement of the service quality and inclusivity, as you know in our country many strategic plan come from above, so planning's that goes from bottom to top are centered on the resource, so it is different to have a planning that from a health center demand and other type; if you base your planning like in many developed countries the low level, or the users, first of all there will not be any wastage and outflow of resources, for example if we plan to receive five types of medicine, but the plan from the top say ten, it means there is a wastage of five. In the same way if we participating the patients and their caregivers, in the planning, and even in the future research, then our plans would be very effective and problem solving. Therefore, their participation plays a significant role.*  ***Health Center Head ID5*** |
|  | ***Awareness raising and service promotion***   - *Awareness creation* - *Mental health promotion* | *I. So, in what extent could the participation of patients and caregivers contribute for the implementation of the project's objective?*  *P. The awareness creation even only could have a big contribution; they are service users, if they are aware of the service and knows where to go first, I think the success of the implementation will be quick....uhh... because they understand this problem as an attack of evil sprite but if they can identify what their problem is and knows where to go to find the solution then I don't see the problem to success.*  *I. So, how could their participation contribute for the improvement of service quality?*  *P. It has a wider contribution. Because the first and foremost they are the starting and the ending points,….uhhh…. so if the community is aware of the cause, the effect, the prevention and the precaution of the illness then they can control it.*  **Health Center Head ID6**  *I. Do you think patients/caregivers should participate in such programs or how important their participation could be?*  *P. They should participate because they are the part of the society and also they are more discriminating patients due to their less awareness, so it will help the patient if families get knowledge, it will help the families to understand mental health problem as a sickness like other types of sickness (not to think it is like evil sprite).*  ***Health Center Head ID8***  *I. Do you think, patients can contribute (after they get recover) by explaining for the society about the sickness and change their perception?*  *P. Yes, they do; I think they can contribute more than any one; first they got better because of the treatment not only this if that person get changed or get better, people in that surrounding (around that person) will know/be aware of what to do when they see other people with this problem.*  ***Policy-maker/planner ID8***  *I. The one who faced the problem is the one who know the difficulty, so, you knew the problem very well and the difficulties you face, then what are you going to advice the planners or what contribution could you have?*  *P. Our contribution will be...we will tell other patients and caregivers to go to hospital...uhh...I use to tell everyone to go there and get treatment...there are many people with this problem on the streets and who are walking around...uhh...people who can be better if they have caregiver...I use to think like this about them...but I am not letting my son to go to street until the day I die, I am going to let him get treatment and I have a hope that he will be fine.*  ***Caregiver ID8***  *I. Besides the health care professionals, the main target of the program is health of mental health patients, who are the main victims and their families/caregivers, So do you think all programs and plans concerning mental health should include the patients and their families/caregivers? and what change do you expect from their participation in the planning?*  *P. Yes! It is good to involve them, because the family members can understand everything, and if there is good result they will be witness to the affectivity of the program. That will help to develop trust among the society.*  ***Health Center Head ID7*** |
| **Need for capacity building** | **Enabling community structures and past experience**   - *Existence of social structures for awareness creation* - *Existence of experience how to select representative* - *Experience from other health care user* - *Experience from other health care user associations* - *Experience of public involvement in health system* - *Right to be involved* - *Value SU/CG lived /experiential knowledge* - *Valuing SU/CG involvement* | *I. Who do you think has to give the training?*  *P. There are trained health workers every were, they can provide the training in different social gathering during "Leqso" and "edir"; "Leqso" is a social practice that if someone from the community died members of the community and relatives come together in the house of the departed for certain number of days in order to comfort the family of the departed: (it is a kind of social mourning therapy); such situation are very good opportunities to meet all members of the community, "edir" is a social support associate also important social gathering.*  ***Caregiver ID10***  *I. You need a person who can raise your voice at planning process like there should be a service; it should be delivered like this and so on, so how would be this person selected to be there? How are you selecting the members of development army?*  *P. In the development army, we usually elect a wise person who is not selfish, who can administer community resource properly and serve the society.*  *I. Then, how can we select patients and caregivers who can represent all mental health patients?*  *P. A person who has a better thinking/understanding and from the patients a person who recovered well, who can explain things clearly and who has better educational level*.  ***Service user ID9***  *I: What about the national mental health association?*  *P: We didn’t meet and discuss about this issue. Do you mean like disable people’s association?*  *I: What is that?*  *P: Disable people are organized themselves and the government helps them to support their living. Actually here there is a big difference between disable people and mentally ill people. A disable person may have a problem on some part of his body but a mentally ill person has a problem on his brain, the main part of his thinking. So it is difficult for us to form an association.*  ***Service user ID5***   1. *Yeah, in some countries there’s really big involvement of patients themselves and/or caregivers as partakers in planning and evaluation and monitoring of the mental health services. Does the ministry have any of that plan to involved patients who recovered from this who are in good state or caregivers in terms of planning for menta health [Crosstalk]* 2. *Mental health. There is a model, but I’m not so sure whether this group is included or not because we’re involving the public wing. We have a wing known as public wing, and it is composed of different associations. One of the associations is professional association, the Midwifery Association, Pediatric Association and things like that, and the other association is the Customer Association. For example, there are patient associations like the Diabetic Patient Association, the Cardiac Patient Association, things like that. So we are involving those people and those groups, but I’m not so sure there is an association for mentally ill and recovered people.*   ***Policy-maker/planner ID*4**  *I: So have you had any experience in Ethiopia, are you aware of any kind of model of that type? Not necessarily in mental health but of caregiver or patient participation?*  *R. I don't have much familiarity about this, but I'm aware that in HIV/AIDS there is a certain involvement of users. And in fact, they represent everywhere, they represent a strong association, usually people living with HIV are much stronger than association of people with mental disorders which are nonexistent or voiceless in any event.*  ***Policy-maker/planner ID*6**  *I. Do you know Tesfa Goh ...which is HIV patients association?*  *P. Yes*  *I. Just like that, there is mental health society in Addis Ababa, what things do you think should be done by this organization?*  *P. It has to work widely*  *I. How?*  *P. Like HIV*  *I. What are those things which have been done by HIV associations?*  *P. About HIV, they are doing many things in different organizations, in health facilities and also on media (television); so, they need to do better than HIV, you know mental illness can be treated like HIV, therefore, it needs everyone's participation.*  ***Service user ID11***  *I. …and the current plan is to integrate the mental health care service into the primary health care centers, so do you think it is workable to your advantage?*  *P. Yes, it will bring change*  *I. How did it bring change?*  *P. For example, previously the nurses were so ignorant, they even don't consider the pregnant woman who is about to deliver, but now there are a lot of improvements, with many meetings and discussions, and we have also made significant contribution by giving our opinion, now we are treated properly, with good respect and dignity, in every hospital or health center, this is the result of continuous discussion and meetings and our contribution of forwarding our feelings and opinions, and sometimes we send delegates and to discuss on behalf of us, representing the whole society. Today, no professional in the hospital or health center mistreat pregnant woman, or they don't let them wait for long or transfer them inappropriately, because we told them not to do this. And just like that I believe if we do the same for the mental health, we can bring change, if we discuss with the relevant people on the issue with agendas, we can change the mind of those who did not understand the situation. If we speak for the mentally retarded people, so people will definitely understand who is expected from them, the effect of one person on the other one.*  ***Caregiver ID9***  *I: But if that were possible, do you think that could be constructive?*  *R: Not could be, it should be. Unless you involve the users, unless you involve the beneficiaries, how do you know? For me, it is very, very critical. And some day it is going to come, but it requires awareness, organization and stuff like that. I think it is very important.*  ***Policy-maker/planner ID1***  *I:Yeah, that would be fine – ]*  *So the question we had was involving patients and caregivers in planning, and so this development and even monitoring the quality of service. So you did mention about having family members as part of the NIMH.*  *R: I did.*  *I: So how would you see that? We don't do that at the moment. I'm not aware of that. Do you think there's potential for that? Do you think it could work in an Ethiopian setting?*  *R: Well, yes, because in the Ethiopian setting, most of the rehabilitation, if you call it rehabilitation, or most of the recovery support is given or done by family members. So whether it's severely mentally taken say to Shenkora holy water or anywhere in the traditional religious healing centers, most of the work is done by the family members, so they have to be involved. So our big problem these days is we bring patients to hospital. Say, for instance, substance abuser or substance-dependent person with alcohol, for instance. So we treat that individual and in hospital, detoxify him, and keep the person for two-three weeks in the hospital. We send him back. So when the person goes back to the community, he's still considered as a drunkard even without taking a drop. So the community has to be involved in the treatment of the individual*.  ***Policy-maker/planner ID*5**  *I: How would you feel about working with patients and caregivers in this way?*  *R: I would be very happy if I can work with them. Do you know what, they can give you actual experience. Responses which are given by Patients and caregivers will be an input for your work.*  ***Health Center Head ID3***  *I. Do you think the participation of patients and their caregivers for improvement of quality service, reliable service delivery and planning of mental health service is important?*  *P. Yes! it is very important.*  *I. Can you give specific demonstration about it?*  *P. First of all, the participation of the patients and their caregivers, gives us about the extent of our service, there feedback is very important, just like if someone is providing service to a certain part of the community, and they return him feedback about the extent of the service they are receiving from that person. second of all sometimes the person who is diagnosing the patients may make mistakes, if the professional make rarely on diagnosing that leads to mistake in the treatment, then weather the person recovered from its illness and what has happened to the patient is only understood from patients and their caregivers, so that is important*  ***Health Center Head ID5***  *I: Do you think participating patient and caregiver in planning is important?*  *R: In my opinion, it is good to participate. Especially, Participating caregivers in medicine supply and other planning is important. People at the grass root level should be convinced on the prepared plan otherwise we cannot achieve our goal. We also have to see the challenges that we may face during implementation.*  ***Health Center Head ID1*** |
|  | ***Mental health advocacy***   - *Empowering stakeholder to involve* - *Advocacy to overcome mental health stigma* - *Need to equip with training* | *I: So how would you see that? We don't do that at the moment. I'm not aware of that. Do you think there's potential for that? Do you think it could work in an Ethiopian setting?*  *R: ….So the community should learn then how to accept a person with mental illness, the community should be empowered how to support that individual, whether it's in establishing a family or whether it's going back to his or her farm, or former business or trade, whatever, or even in teaching the person to learn new skills and assimilate into the community. So the community is very important. So if we have a national institute of mental health that specializes in the community support, doing research, giving training to the community, and using models, how to support chronically mentally ill individuals in relocating those individuals to the community. If you have national institution of mental health, that means you have different departments. So departments that specialize in the community support, department that specializes in women's issues or children's or youth issues, so we need to have different departments that focus on different issues and address different issues.*  ***Policy-maker/planner ID5***  *I: But what about would you see a role of family members or patients at sort of a more high level, like planning, being involved in decisions about how we develop the services, how we monitor them at a kind of higher level. Would you –*  *R: Well, higher level may be too much, but if we kind of bring that kind of service at the lower level, that base of the society. If you look at the pyramids, that's the base, right? [reference to WHO pyramid] So if we empower the base, then for a high-level policymakers life becomes much easier because in the end of the day, it's the base, the community that does much of the job.*  *I:And we've got a caregiver organization in Ethiopia, the Mental Health Society.*  *R: Yeah.*  *I: We don't really have a patient organization as such. How do you see it? Do you think that could be a contribution or –?*  *R: Again, if we start at the lower level, that may take you to one state, for instance. So you have Tigray. You have Mek'ele, the capital, and then… Mekele. You take one neighborhood, one kebele [administrative sub-district]. So if you empower that kebele [sub-district] say with a population of 5000 or 10,000, so it would be much easier for those kebeles [sub-districts], that neighborhood, (a) to identify someone early with mental health issues, whether it's depression, dissatisfaction in life, serious psychiatric problem, the form of depression or psychosis, or anxiety or whatever, as a result of say some life event. So it's those kebele individuals who can come in first detect the behavioral issues on that individual. So if you kind of empower that small kebele, then most of the job is done. They can early detect mental illness and bring the individual to the nearby health service. So they can financially support, emotionally support, spiritually support, or – that's what is actually done, actually. But now even if the kebele dwellers know that there's something wrong with an individual, they don't want to dwell on that, because they'll say, "Oh, what if he or she rejects our offer?" But now if we kind of train them and properly empower them, and then I don’t know how many woredas [districts] we have in this country. We have over 300 woredas in this country. So if we empower each woreda, train them properly and show them how to support those individuals through the help of these health extension workers, because we shouldn’t leave everything to health extension workers because the community has to be properly empowered. After all, it should be the responsibility of the neighborhood 'cause if they see someone who is lately drinking with the binge drinking, for instance, it should be the kebele elders who should come first and support that individual. 'Cause after all, the health extension workers has no power on every individual in the neighborhood.*  ***Policy-maker/planner ID5***  *I: Okay. Thank you. And maybe another approach people could say is that if service users and caregivers were more empowered, they might also be able to kind of push to ensure safeguards. I don’t know. How do you see that whole area and sort of patient –*  *[Crosstalk]– empowerment, service user empowerment and involvement?*  *R: I'm truly speaking very keen on that. I like the idea that service users are to be empowered and have to have a role in contributing to – yeah. So I think that depends on how open and receptive at different levels we get decision-makers and service administrators. Because not just for mental health, for other aspects of health and other services in general, I think the government of Ethiopia tries to engage people by different public forums, for instance. So users are getting encouraged to form groups. And, also administrators and leaders are encouraged to, also, communicate with groups of customers/clients from different aspects of life in contributing to the health, improve the service, and also to also raise their concerns and issues. So they need to be involved in the decision-making process as well. So I think this is an opportunity for mental health, but the practice is going to depend on the attitude of leaders and administrators in general. But interest needs to be also stimulated at the ministry of health level so that it can influence the different administrators. But based on my experience in Amanuel, fir instance in the hospital I work, patients are getting more and more empowered in my opinion, through the process of rehabilitation and also better engaged with their people who deliver service and different dynamics so that we're now having a group of patients who are helping us to improve the hospital compound by gardening, by also forming different groups. Just this morning, they were suggesting to me to start the transitional house for our addiction patients, and they have already started collaborating with interested groups to rent houses and then help provide such services so that people who get detox and do not necessarily have to get back soon to the community, but the houses there during that transition period can be useful. So these are the kind of things they can easily communicate with us now. And there are also artists who have been former addicts and mental health service users now are also suggesting to us to produce different documentaries of mental health using prominent actors in the country. So I hope soon these things can happen, and these ideas come because mental health service users are now trying to engage at a different level as well to help us see the different aspects of their needs and not just of the mental health service alone. So I think these are the kind of things that we'll continue to build on and improve the dynamics of engagement between service givers and service users, I think. But it's very important, very, very, very critical In fact. They have to be empowered.*  ***Policy-maker/planner ID7***  *I. Do you? Do you think patients/caregivers participation can contribute to achieve a better result on minimizing stigma?*  *P. Yes, their participation is very important, because families may not understand the problem and/or they might not think its treatability, even though they don’t stigmatized patients because they (families) are their families; but there are cases which families take patients to different hospital and holy water by understanding what their problem is. So, their participation is very useful for the society because families can explain if they face stigma and related problems.*  *I. Can they explain?*  *P. They can explain for people in that community.*  *I. Do you think, patients can contribute (after they get recover) by explaining for the society about the sickness and change their perception?*  *P. Yes, they do; I think they can contribute more than any one; first they got better because of the treatment not only this if that person get changed or get better, people in that surrounding (around that person) will know/be aware of what to do when they see other people with this problem.*  ***Policy-maker/planner ID8***  *I. But do you think this is really what is happening practically? Do you think people are positive when they are getting improved?*  *P. I mean, it is not the same with everyone, it depends from one person to other person, that is why we need to teach the people, we have to tell the people that the patients are part of our society family and village,*  ***Caregiver ID10***  *I. As I told you PRIME is working on service delivery and EMERALD is working on health system development, therefore, how could patients and caregivers’ participation improve the mental health care implemented in your district through the PRIME project? How much is their participation important for the achievement of its objective?*  *P. It may have a vast contribution.*  *I. Can you explain it please?*  *P. For example, as I told you before, previously people used to consider them as mad person (unable to recover)...uhh... but, the survey which recently conducted at the health center level disproved that...they asked to identify people with this problem and they gave treatment, therefore mobilization is the crucial aspect.*  ***Health Center Head ID6***  *I. What should be done for all of you regarding this?*  *P. The main cause of this problem is our socioeconomic problems, like thinking a lot about many things, thinking about how to overcome low standard of living... for example, in my case I had been working a daily labour work to raise my child and one day I fight with one person he was a civil servant, and I hurt him badly and the police ordered to catch me where and whenever they find me , they found me after two and half years and they bit me severely, my sickness starts since then. During that time I was very worried about raising my child which I had at the old age or after I spend most of my age in the army; so, the main causes are the above mentioned things and the solution can be if the government can provide house for those of who don’t have a family to support them and also if we can get organizations which can help us like they are doing for HIV patients.*  *I. Are you saying financially?*  *P. Yes!*  ***Service user ID9***  *I: What kind of training might help you to work with patients and caregivers?*  *R: To work with them you have to have better knowledge. This is because they may raise their real experience since they are living with the problem. They may ask you actual issues which they face when they took the medicines. If you can clarify them they will build confidence to work with you. You need to know more. The kind of training that helps you to work with people and help you to convince others are the important once.*  ***Health Center Head ID3***  *I: What kind of training do you think is important to involve patients and caregivers?*  *R: Here everybody should know his right and responsibility. Sometimes one body may ask his/her right without working on his responsibility. If stakeholders do their work we can improve quality of service.*  ***Health Center Head ID1***  *I: What type of training might help you to work with patients and caregivers in this way?*  *R: Communication training like how to communicate with them, ways of communication and at what communication level is important. When you involve them, they became your helper.*  ***Health Center Head ID4***  *I: What kind of training do you think participants should get?*  *CG: They should get training on how to take care of patients.*  *I: I think you are telling me for all caregivers who came for the health service. What about for the caregivers and patients selected to participate in planning health service and researches?*  *CG: In my opinion since there are many mental health problems participants should get trainings on different mental health problems for example, on how to take the medicines; on how to give care for patients; how to collect the required information timely and in efficient way. So giving training is mandatory.*  *I: How important do you think giving trainings for patient and caregiver participants?*  *CG: Training is mandatory. The participant should know even how to work, what to do and how to behave with colleagues in the team.*  *I: What kind of training do you think is important?*  *CG: A training that helps participants to improve their capacity in working with team, the type of work they are going to do and how to do their future work.*  ***Caregiver ID3***  *I. So what type of specific training do you think is you need to work in such situations, like working with the mental health patients and their caregivers?*  *P. First of all community system, sociology type of trainings to understand the society better, because we only know heath related things. And on how to make contacts with the main bodies of the project, as well as short term trainings on this specific health matters. Otherwise we don't need to repeat what we already know.*  ***Health Center Head ID5***  *I. What type of training do you think you need?*  *P. General knowledge about mental health, and "Sensitivity" training, which is about the impact of the problem for the society and its wellbeing, such trainings, is very helpful to work with our community. If we understand those issues then we can teach and organize the community.*  *I. What type of training or facility do you think should be given to you so that you can work with the community, with the patients and their caregivers?*  *p. We must know about the mental health problem very well, so that we can explain in detail, we may teach in the every-morning health education at the health center. ..uhh...*  ***Health Center Head ID7***  *I. What type of training do you think is important, if you are allowed in the evaluation and control of all these, and the implementation of the service?*  *P. Yes! We need training. We need to know what we have to do, at all level, so that we will have acceptance by the people whom we are going to work together. We need to know the regulations rules related to our responsibilities.*  *I. What is the importance of knowing the rule and regulation for your participation?*  *P. Everything is according to the rule and regulation, we must know what we have to do and what we don't have to. We must know the extent and the limits of our right and what duties we have to carry out.*  ***Caregiver ID10*** |
|  | **Service user and caregiver mobilization and empowerment**   - *SU/CG organization and representation* - *Space and structure for involvement* - *Treatment and more service in addition to medicine* | *I. We have said that the opinion of mental health patients, their families and caregivers is highly needed in order to be considered during health care planning, but they need to be supported by certain type training which will enable them to identify their problems in detail, and methodologies on how to advocate for the wellbeing of the patients, therefore the basics of the training are to built the capacity so that people can speak out their problems?*  *P. In that case my first recommendation even before giving them financial and any material support is establish a kind of club, where they can discuss together, people of the similar cases should come together in one place, they must built friendship among themselves, then education can be conducted on different subject matters, along with the training different types of discussion could be developed and in this way we can provoke them to stand for their right and the rights of patents. In this way e can also reduce the problems*  *I. Do not you think there will be sustainable solution?*  *P. I don't think there will be a sustainable solution, but what I think these people [mental health patients], is proper acceptance and treatment, if they have good medication and if they are provided with conditions that may develop their thinking they can be improved, they can be even functional, what they need is good treatment*  ***Caregiver ID10***  *I: What is your view on that in relation to expanding mental health care?*  *R: I think is wonderful like in the …, I had worked in the director of several programs and it was mandatory to work with the user group, the advocacy group. But it takes a lot of sophistication to do that especially… what we have is the national Ethiopian mental health society. Very weak. The families. But, you know I do believe, definitely, it is also our responsibility to help them organize but we are not doing a good job. We are not doing anything actually.*  ***Policy-maker/planner ID1***  *I: That may be right. But you may say somebody who is in this condition of health, who has the capacity of speaking in front of others.*  *P: You said from patients, right?*  *I: Yes, from patients.*  *P: But when the coordinator call us (from the hospital), we will come. He can make the organization.*  *I: Yes, that is true. My question was do you have anything to say to be improved on the researches done before?*  *P: Yes, I have.*  *I: What is that?*  *P: Thanks to God, you are doing well. It is good to organize patients and create conducive environment to have discussions within themselves*  *I: I have completed my question. Do you want anything to add?*  *P: As I told you before it is good to organize mental health patients. This would allow having discussions within themselves or with other officials. At the same time we can recreate and have fun*  ***Service user ID6***  *I. Do you know Tesfa Goh ...which is HIV patients association?*  *P. Yes*  *I. Just like that, there is mental health society in Addis Ababa, what things do you think should be done by this organization?*  *P. It has to work widely*  *I. How?*  *P. Like HIV*  *I. What are those things which have been done by HIV associations?*  *P. About HIV, they are doing many things in different organizations, in health facilities and also on media (television); so, they need to do better than HIV, you know mental illness can be treated like HIV, therefore, it needs everyone's participation.*  *I. So, what can they do?*  *P. What they have to do is education, training ...as I told you*  *I. For who?*  *P. For the society?*  *I. What type of education for example?*  *P. For parents...I told you a person should not be stressed...stress caused when a person thinks beyond ... and also they should tell for a married women where she can get solution if her spouse or how her marriage bring her happiness... as well as where to get legal advices.*  *I. First what things can be changed if you or other patients can participate in the planning process, better changes can be seen than the existing?*  *P. There might be a change if patients can be organized*  *I. How?*  *P. If they can organized under an organization/association and get education and training and then the one who took the training should teach the others.*  ***Service user ID11***  *I: What do you think if you organize yourself and form an association? That may help you to request your rights and improve the service delivery.*  *P: Yes.*  *I: Why? What do you think is the benefit?*  *P: For many things.*  *I: For example, what kind of benefit?*  *P: It shows our improvement and refreshes our mind.*  *I: You may not get good service. Do you get an opportunity to tell your compliant?*  *P: By organizing ourselves.*  *I: How?*  *P: By organizing ourselves and contributing ideas we can bring changes.*  ***Service user ID7***  *I. So, what things should be done to increase your participation?*  *P. for us*  *I. Yes, I think all of you might not be capable to participate, so, to make you or others able to participate …what I mean is what type of support do you need?*  *P. Teaching and telling them the strategy properly*  ***Service user ID12***  *I. For example, you are receiving the service, but the service needs monitoring, so if you or caregivers work with the controllers…what are you going to tell the controllers to correct?*  *P. To get our health back and to be beneficiary, they should tell us the rules and regulation from the government and also things we should do not only to oppose their work.*  ***Service user ID10***  *I. What type of training do you intend to receive?*  *P. In a regular manner, by getting together and set a plan once in a month or in a two month in appropriate place by appropriate person.*  *I. What do you need?*  *P. Daily programs.*  *I. What do you expect to learn during the program? What do you need to know and to ask for your rights in this regard?*  *P. What we need to know is that the day of the educations, where everyone is gathered, and then we can also gather the mental health living in our neighborhood, and at that time we can also choose people who can represent us, people that can speak on our behalf.*  ***Caregiver ID9***  *I: What else do you think should be done to improve the active involvement and participation of people with mental health problem and their caregivers in such undertakings?*  *CG: Care is very important if for example the professionals believe you are getting better …they could neglect the patient if she/he isn’t alert and active to ask for a proper treatment. So it is important to have people who know and speak on the patients in such instances.*  *I: So that they give the patient the appropriate treatment?*  *CG: Yes, they usually do that if they think the patient is better and another patient comes, so if there is a person who can speak about this and other problems on behalf of the patients and participate in health care service quality control activities. I believe we will able to get a better service*  ***Caregiver ID4***  *I. What about selecting delegates from the patients, how can we identify delegate from the mental health patients?*  *P. To select the good one?*  *I. Yes! How do you think the selection can be done?*  *P. This cannot be worked out be voting or asking recommendation, not by the opinion of family members or kebele authorities; it must be by appropriate and relevant researching, because they may do this for favoritisms, or without knowing the objective.*  ***Caregiver ID10***  *I. How it can be useful if there is an association?*  *P. I think if there is an association it will help them to take their medication properly and on time together.*  *I. Is it helpful to take medication only?*  *P. No, there are many other things.*  *I. Please tell me*  *P. What I mean is , if a person starts taking medication, at the same time that person will learn about many things.*  *I. How?*  *P. For example, that person I going to be questioned about how his/her sickness starts and also they are going to ask what that person is doing for living...that will help.*  ***Service user ID12***  *I. So, what kind of change it will bring?*  *P. To bring change, we should get together and discuss about solutions and things which are helpful for us.*  *I. For example, what?*  *P. Anything we are using...uhh…we are mentally ill people, so anything you or health professionals provide us; we are going to use if we think it is useful for us or we are not if we don’t think…uhh… but still we are taking medications which they are giving us, they are helping us as much as they can.*  *I. What kind of training do you need to be able to go forward by yourselves?*  *P. We should be together, we need to stay together that we couldn’t stand problems related to our sickness, so, we need to organized or we should establish our own unity; we want our health.*  *I. So, what things should be done to make you able to fight for your rights together?*  *P. We will discuss with them, we should explain things whenever we meet them; because thanks to Him [God], we are fine if we take our medication and we are sick if we don’t. All of us don’t have equal standard of life somebody may have good life and the other may not; the medication use to help us if it has vitamin, if not it will hurt us because we have only Kocho to eat, only a person who can get good food can resist the medication*  ***Service user ID10***  *I: So, mental health is the concern of everybody. In your area, when you made discussions with the woreda officers or heath extension workers about health service improvements for other diseases, you propose things to be done or improve? Likewise what do you think to be improved here in mental health area?*  *CG: If government didn’t give medicines for mental health patients, things will become difficult. The medicines made patients to calm down.*  ***Caregiver ID1***  *I: From your experience of mental health, what kind of contribution can you made?*  *P: After I get cured from my mental health problem, I will make what is expected.*  *I: What about now while you are following your medication?*  *P: Now I don’t have money, I am following my medication by the help of other people. I am coming from far place Medhanialem.*  ***Service user ID1***  *I: My idea is …well mental health patients with their care givers come to this and other health care centers to get mental health care services. So at times they could meet people such as we who come from different universities and institutes to do researches on issues related with mental health problem and mental health care services. So they may ask the patients and the care givers for some information or some questions. So the main purpose of these researches is to improve the quality of health care service in the future. So what do you think about this? They get the treatment they came here for but at the same time they also actively participate in the researches. So what do you think should be done to ensure this? To ensure the active participation of people with mental health problem and their caregivers in such researches? We believe that it is very important to have their ideas to be able to deliver a health care service with a better quality in the future.*  *CG: Well, in order to have their active participation, I think it is important to have them take their medicines properly. It is also important to aware them, get them to discuss with one another on matters that concerns them and learn from one another and enhance their motivation.*  ***Caregiver ID4*** |
